# Intramammary Tumor Location and Ipsilateral Lymphatic Spread in Early Breast Cancer Patients Using One-Step Nucleic Acid Amplification (OSNA) Assay

**DOI:** 10.1155/2024/4864443

**Published:** 2024-11-08

**Authors:** Mariana Robalo Cordeiro, Inês Gante, Daniela David, Ana Gomes, Margarida Figueiredo-Dias

**Affiliations:** ^1^Gynecology Department, Coimbra University Hospital Center, Coimbra, Portugal; ^2^University Clinic of Gynecology, Faculty of Medicine, University of Coimbra, Coimbra, Portugal; ^3^Pathology Department, Coimbra University Hospital Center, Coimbra, Portugal

**Keywords:** breast neoplasms, lymph nodes, neoplasm, OSNA, prognosis, site

## Abstract

Establishing an accurate prognosis for women diagnosed with breast cancer (BC) is extremely challenging. Axillary lymph node (ALN) evaluation is considered of major prognostic value. The one-step nucleic acid amplification (OSNA) assay is currently used for assessing axillary sentinel lymph node (SLN) status in BC. Additionally, total tumor load (TTL) may help predict further metastatic axillary involvement beyond the SLN. The prognostic value of primary BC location remains controversial due to lack of consensus on the biological differences among tumors at various sites. Evidence suggests that tumors in the internal quadrants (INLs) have worse prognosis compared to those in the external quadrants. Furthermore, ALN involvement is believed to be mainly associated with external quadrant tumors, mainly due to the lymphatic drainage system of the breast. This pilot observational study, despite lacking a control group and having a relatively small sample size, is the first to evaluate the potential relationship between primary BC location and ALN metastasis using the OSNA assay. A sample of consecutive BC patients undergoing axillary staging with the OSNA assay were included. Tumors were categorized into three groups based on primary location: external quadrants and axillary tail (EXL), INLs, and nipple and areola location (NAL). Although not statistically significant, the INL group exhibited a higher mean TTL. Additionally, no significant differences were observed between groups concerning SLN detection techniques, SLN status, number of metastatic SLN, or mean TTL. These findings support the use of the innovative tracer superparamagnetic iron oxide regardless of tumor site. This study underscores the importance of understanding the relationship between BC location and ALN status, which may improve prognostic stratification and targeted therapies based on tumor site. If these observations are confirmed in larger, multicentric studies, the potential conclusions may shift the paradigm of INL tumor treatment, significantly impacting clinical practice and research.

## 1. Introduction

Establishing an accurate prognosis for women diagnosed with breast cancer (BC) is extremely challenging. Among many factors linked to BC survival, axillary lymph node (ALN) evaluation is considered of major prognostic value.

Sentinel lymph node (SLN) biopsy is the standard surgical approach for pathologic nodal staging in patients with early BC and clinically negative axilla [1, 2]. According to the most updated guidelines, only patients with three or more positive SLN are systematically further submitted to axillary lymph node dissection (ALND) [3, 4]. The conventional pathologic examination of frozen SLN sections has a substantial false-negative rate of up to 30% [5, 6]. For this reason, the one-step nucleic acid amplification (OSNA) is currently being used for the assessment of axillary SLN status in BC [7, 8]. This assay quantifies the cytokeratin 19 (CK19) mRNA copy number in the lysate of SLN, which carries several advantages as it provides objective and semiquantitative information of the whole SLN in a short time, without much effort by pathologists and with low interobserver variability, making it a reproducible and standardized method [9]. Nevertheless, one of the major pitfalls of the OSNA has been considered the destruction of the SLN, which precludes further analysis [8].

More than a decade ago, Tsujimoto et al. established OSNA cutoff levels for SLN biopsy results interpretation, defining macrometastases as > 5000 copies/μL of CK19 mRNA, micrometastases as 250 to 5000 copies/μL, and absence of metastases or presence of isolated tumor cells (ITC) as < 250 copies/μL [10]. The total tumor load (TTL), defined as the total amount of CK19 mRNA copies in all positive SLN, seems to help predict additional metastatic axillary involvement besides SLN, especially because TTL is independent of the number of metastatic SLNs [10]. Even though there is no consensus on the exact TTL cutoff levels to determine ALND, some authors have been discussing this issue. In 2013, Peg et al. have proposed a preliminary TTL cutoff value (TTL > 15,000 mRNA CK19 copies/μL) for ALND decision [11]. In the PORTTLE study, in 2020, Fougo et al. established a relationship between TTL > 30,000 mRNA CK19 copies/μL and the presence of nonsentinel ALN metastases, considering this cutoff value safe to aid the decision of further ALND [7]. More recently, Kenny et al. observed that a TTL ≥ 110,000 mRNA CK19 copies/μL and lymphovascular invasion (LVI) can strongly predict metastasis in four or more ALNs [12, 13].

Primary BC location prognostic value is controversial, mainly because there is lack of agreement on the biological differences among tumors of different sites [14–17]. However, there are studies suggesting that tumors located in the INLs may be associated with worse prognosis, in terms of distant metastases and survival, compared to those confined to the external quadrants [15, 18–22]. This evidence seems to be supported by the lymphatic drainage observed in these tumors to the internal mammary lymph nodes, which is not consistently evaluated [23]. Until now, it was believed that tumors confined to the external quadrants have a higher tendency to involve ALNs comparing to tumors of other locations, mainly due to the reduced tumor-to-axilla distance [14, 15]. However, recent data suggest that lymphatic drainage from different quadrants of the breast may not follow exclusive paths [15, 18, 23]. The OSNA assay, especially with the additional relevant information from TTL values, paves the way to a more accurate and quantitative axillary staging, which can make a real difference in stratifying breast tumors according to their location and likelihood of SLN metastasis, for both the axilla and the internal mammary lymph nodes.

Understanding the relationship between primary BC and axillary nodal status is an important research area that could enhance prognostic stratification and assist in tailoring adjuvant therapies, including the avoidance of axillary dissection and its inherent morbidities. This understanding may also contribute to the development of future targeted BC therapies based on the location of the primary tumor.

To date and to our knowledge, this is the first study aiming to evaluate the potential linkage between primary BC location and ALN metastasis using OSNA assay.

## 2. Materials and Methods

### 2.1. Study Design and Participants

This study is a retrospective, cross-sectional, observational study. It was performed according to the recommendations of the seventh revision of the Declaration of Helsinki on medical research involving human subjects. Upon approval of Coimbra University Hospital Centre Ethical Committee, patients were given an informed consent to be included in the study. Individual medical records contained in the electronic patient registration system were consulted for each included patient, and data anonymization was performed.

Consecutive patients with early BC submitted to axillary staging with OSNA assay were included. The exclusion criteria were defined as male sex, age under 18 years old, pregnancy, neoadjuvant treatment, tumors not expressing CK19, multicentric or bilateral tumors, and patients unable to give informed consent.

Tumors were divided into three groups based on primary tumor location, such as external quadrants and axillary tail (EXL), INLs, and nipple and areola location (NAL).

### 2.2. Study Variables

The main variables of the study were the SLN status (positive or negative), the SLN OSNA result (pN0, pN1mi, and pN1), and the TTL (copies/μL of CK19 mRNA).

The clinical stratification factors as patient's age at diagnosis, parity, breastfeeding, menopausal status, postmenopausal hormone therapy, tobacco, and palpability were included. Pathologic factors, such as tumor largest diameter, nuclear grade, histologic type, LVI status and Ki67 index, were considered. The intrinsic molecular subtype defined as Luminal A, Luminal B, Luminal B/human epidermal growth factor receptor-2 (HER2)+, HER2-enriched or triple-negative, evaluated by the expression of estrogen receptor (ER), progesterone receptor (PR), and HER2, based on the 2021 St. Gallen guidelines were also included. The technique for the SLN detection, such as the superparamagnetic iron oxide (Sentimag®) and the combination of patent blue dye and radioisotope (Technetium-99m), was also considered for stratification.

### 2.3. Statistical Analysis

Data analysis was performed with SPSS software, Version 28. The Shapiro–Wilk test was used to assess the normal distribution of quantitative variables. Sample description was made with measurements of central tendency and dispersion (minimum, maximum, and mean [± standard deviation (SD)]), and frequency tables. To compare independent and paired samples, parametric (independent-samples *t*-test and one-way ANOVA) and nonparametric (Fisher exact test or chi-square test) tests were performed depending on the variable type and distribution. Statistical significance was set at *p* < 0.05, for a confidence interval of 95%.

## 3. Results

A total of 235 patients with BC were included in this study and divided into three groups according to the primary tumor location as EXL (*n* = 160, 68.1%), INL (*n* = 58, 24.7%), and NAL (*n* = 17, 8.4%). There were no statistically significant differences concerning clinical characteristics between the three groups, which are presented in Table 1. However, the EXL group showed higher percentages of smoker patients and palpable tumors.

Pathologic characteristics of the tumor according to location are shown in Table 2. The majority were no special type (NST) and Luminal A tumors. Even though without a statistically significant difference, the INL group showed a higher percentage of LVI. There was a statistically significant difference in tumor grade between groups (*p* = 0.048) with a lower grade observed in the EXL group (versus in INL and NAL groups; *p* = 0.021). However, there were no statistically significant differences concerning the remaining pathologic criteria such as histologic type, tumor diameter, LVI, Ki67, or intrinsic molecular subtype (Table 2).

A summary of the main characteristics of the SLN according to tumor location is presented in Table 3. There were no statistically significant differences concerning number of metastatic SLN, OSNA result, and TTL (Table 3). SLN was detected using superparamagnetic iron oxide (Sentimag®) or patent blue dye combined with radioisotope techniques in a similar proportion (*p* = 0.905). The mean number of metastatic SLN detected was almost coincident for each group (*p* = 0.542). Despite no statistically significant difference, a tendency for a higher mean TTL was observed in the INL group (*p* = 0.708).

**Figure 1:**
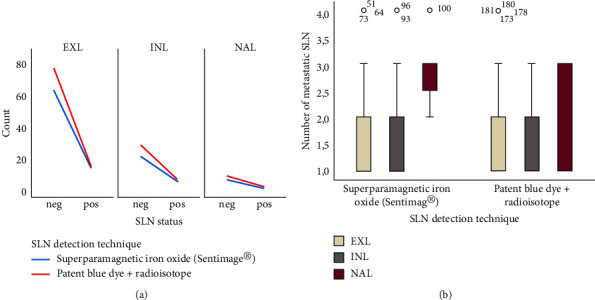
Evaluation of the influence of sentinel lymph node (SLN) detection technique in axillary staging among the EXL (external quadrants and axillary tail), INL (internal quadrants), and NAL (nipple and areola location) groups: (a) Line chart representing the frequency of SLN status according to SLN detection technique among the three groups; (b) box-plot graphics representing the spread of number of metastatic SLN according to SLN detection technique among the three groups.

**Figure 2:**
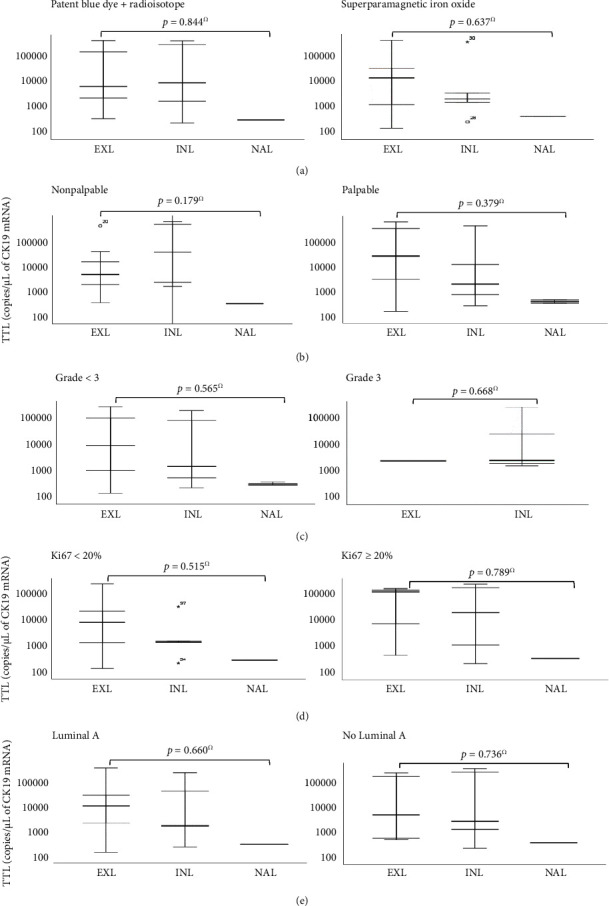
Box-plot graphics representing the subgroup analysis performed to evaluate the association of TTL with different clinicopathologic parameters among the EXL (external quadrants and axillary tail), INL (internal quadrants), and NAL (nipple and areola location) groups: (a) Technique used for SLN detection; (b) palpability; (c) tumor grade; (d) Ki67 index; (e) intrinsic molecular subtype. Ω: one-way ANOVA.

To evaluate the potential influence of the technique used for the SLN detection in axillary staging among the three groups, a subgroup analysis was performed and no statistically significant differences were found between the technique of detection and SLN status, the number of metastatic SLN or the mean TTL (Figures 1 and 2).

TTL has shown to be of the utmost importance in clinical practice, especially for being considered an independent predictor of the non-SLN status, and hence, further subgroup analyses were performed with focus on the TTL. No statistically significant differences were found regarding TTL and clinicopathologic parameters, such as the technique used for SLN detection, tumor palpability, tumor grade, Ki67, and intrinsic molecular subtype (Figure 2).

## 4. Discussion

SLN biopsy is the standard approach for axillary staging in early BC patients with clinically negative axilla, which has long become a less-invasive alternative to ALND. Nevertheless, SLNB still has a non-neglectable false-negative rate in predicting ALN status, even with the OSNA assay. Indeed, novel molecular imaging technologies combined with artificial intelligence predictive models will help to optimize axillary staging interventions, even without requiring SLNB. Hence, it is crucial to evaluate potential prediction factors of ALN involvement.

The influence of clinicopathologic features on predicting the risk of metastasis to the axillary SLN has been described by many authors, but the results remain either controversial or lack in accuracy [24, 25]. Tumor size has consistently been identified as a strong predictor of ALN invasion, as well as age at diagnosis, menopausal status, HER-2 status, nuclear grade, LVI, and Ki67 index [24].

Another important factor to be considered is the primary breast tumor location, which is also debatable, mainly because there is no agreement on the existence of biological differences among tumors of different sites [26]. There are at least nine studies to date that have explored the association between breast tumor location and ALN status. The results of six of these studies suggest that the external quadrant tumors have a higher tendency for ALN involvement compared to tumors from other locations. Indeed, a large study by Manjer et al. in 2004, in a series of 3472 Swedish invasive BC patients submitted to ALND, concluded that tumors in the external quadrants had a higher tendency to invade ALN comparing to the INL tumors [18]. In 2020, a population-based multicentered study of 7680 patients with invasive BC, including 5125 patients with clinical negative axilla that were submitted to ALND, established a positive correlation between tumors in the external quadrants and ALN invasion [27]. Also, Bevaliacqua et al., in a retrospective study of the first 2000 BC patients undergoing axillary SLNB at Memorial Sloan-Kettering Cancer Center, revealed a less frequent association of ALN metastasis in upper-inner-quadrant tumors compared with all other quadrants [28]. Less robust studies, such as the retrospective study performed by Halverson et al. in 1994 with 514 NC patients submitted to ALND and the Fein et al. prospective study with 445 patients, also confirm this tendency of the external-quadrant tumors to more frequently invade ALNs [29, 30]. On the other hand, three studies did not find any statistically significant associations regarding tumor location and nodal status. In the retrospective study of Maibenco et al., which included a large cohort of 12,950 patients with invasive, infracentimetric breast tumors undergoing ALND, no differences were found between tumor site and ALN metastasis [31]. In 1998, a similar conclusion was described by the Velanovich research team in 851 consecutive invasive BC patients who underwent ALND [32]. We should carefully interpret these results because no high-level evidence studies were performed about this issue. Besides, the data available did not include BC patients submitted to SLNB with novel techniques for SLN detection, such as the superparamagnetic iron oxide technique. Nevertheless, tumors located in the INLs seem to be associated with worse prognosis, concerning distant metastases and survival, compared to those confined to the external quadrants [15, 20–22].

The correlation between breast tumor location and lymph node metastasis is primarily based on the lymphatic drainage pathways of the breast, which in up to 90% drain into the ALNs [33]. Therefore, the proximity of the primary tumor to the lymph nodes of the axilla might influence the likelihood of ALN involvement. Indeed, the breast has an extensive network of lymphatic vessels that typically follow a specific pattern, mainly constituted by the ALNs, the internal mammary lymph nodes, and other regional nodes, such as the supraclavicular and infraclavicular lymph nodes [33, 34]. Hence, it is believed that the majority of tumors in the INLs tend to metastasize to the internal mammary lymph nodes in opposite to the ones confined to the external quadrants that preferentially metastasize to the ALNs [14, 20–22]. There is no agreement regarding ALN metastasis incidence concerning tumors located under the NAL. Some authors suggest that the smaller the tumor–nipple distance, the higher the likelihood of ALN positivity [19, 35].

Even though no statistically significant differences were found among the different analyses performed in this study, the TTL mean value was higher in the INL group. This group of tumors has shown some relevant pathologic characteristics linked to poorer prognosis, which might help explain this finding. In comparison with the other two groups, the INL group showed higher percentages of triple-negative intrinsic molecular subtype (3.4%, *n* = 2), of LVI (8.6%, *n* = 5), of two metastatic SLN (3.4%, *n* = 2), and of positive SLN status (11%, *n* = 11). Thus, the INL group tumors appear to have an intrinsic aggressive behavior conferring a higher risk of metastasis.

The higher TTL mean value observed in the INL group seems to be inconsistent according to the preferential involvement of the internal mammary lymph nodes by these tumors. However, recent literature advocates that lymphatic drainage from different quadrants of the breast may not follow exclusive paths and that all parts of the breast can drain to both ALN and internal mammary lymph node [23], [36–38]. Even though there are clinical trials indicating that IMLN metastasis does not independently predict overall survival and progression-free survival for BC patients who receive personalized adjuvant treatment, internal mammary lymph node invasion has been observed in up to 52% of ALN-positive patients and in up to 17% of ALN-negative patients [39, 40]. Yet, there is no consensus about whom would beneficiate from internal mammary lymph node irradiation, especially due to the side effects of this therapy associated with the increased dose-volume of cardiac and pulmonary irradiation, but also because of the depth of internal mammary lymph node location within the chest wall that makes the rate of clinically detected internal mammary lymph node recurrence low [35, 40]. Among other reasons, patients included in this study were not staged for the SLN status in the internal mammary chain, especially because the ones submitted to patent blue dye combined with radioisotope SLN detection received superficial injections of patent blue dye, which is inadequate for this purpose [37]. For this reason, we cannot exclude the presence of internal mammary lymph node metastasis in INL, EXL, or NAL groups that would possibly help in better exploring our results.

The limitations of a two-dimensional (2D) imaging modality evaluation and classification of breast tumor location, such as conventional mammogram, should also prompt careful interpretation of these results, especially because it may not accurately represent the true spatial extent of the tumor, including its depth, and breast tissue overlapping can also occur, making it challenging to precisely determine its location [41, 42]. Furthermore, the three-group division of the study population do not specify the exact quadrant of tumor location, which could have had influence in the results.

This study underscores that the technique used for SLN detection does not influence axillary staging outcomes. Our analysis found no statistically significant differences in SLN status, the number of metastatic SLN, or the mean TTL when comparing the SPIO technique to the combined use of patent blue dye and radioisotope. These findings confirm the noninferiority of the SPIO method relative to the conventional radioisotope technique across various breast tumor locations. The equivalence in performance between these methods suggests that SPIO is a viable alternative for SLN detection. The use of SPIO offers several advantages over traditional mapping agents, including the elimination of radiation exposure, which is particularly beneficial for both patients and healthcare workers. Furthermore, SPIO reduces the risk of allergic reactions associated with patent blue dye and bypasses the regulatory complexities and logistical challenges linked to radioisotope use. This method utilizes magnetic nanoparticles that are detected by a magnetometer, offering precise localization of SLN without the need for radioactive tracers. This is not only safer, more sustainable and environmentally friendly, but also simplifies the logistics of SLN biopsy procedures, as SPIO tracers have a longer shelf life and are easier to handle compared to radioisotopes. The broader implications of these findings are significant. The adoption of SPIO for routine SLN detection can streamline clinical workflows and reduce the overall burden on nuclear medicine facilities. Additionally, the safety profile of SPIO makes it suitable for repeated use, which could be beneficial in monitoring patients over time or in settings where radiation exposure needs to be minimized. In summary, its comparable efficacy, coupled with its safety and logistical benefits, supports its integration into clinical practice. Future research may focus on long-term outcomes and cost-effectiveness to further validate the widespread adoption of SPIO in SLN biopsy procedures [43–45].

All of the evidence found in this study emphasizes the need to understand the presumed link between primary breast tumor site and axillary nodal status, especially with quantitative techniques as OSNA assay, which might improve prognostic stratification accuracy, as well as tailor adjuvant therapies and help the development of targeted therapies based on tumor location. Likewise, the biological behavior of tumors confined to the INLs should merit thorough investigation so that a more precise staging can be performed to improve survival in these patients. Hence, it is critical to develop superior molecular imaging technologies to enhance the knowledge of the genomic and radiomic features of BC patients with high risk of internal mammary lymph node involvement, in order to offer personalized and rational internal mammary lymph node surgery or irradiation strategies. In parallel, the ongoing shift toward less invasive surgical approaches in BC—moving from radical mastectomy to lumpectomy, and from routine ALND to SLN biopsy—represents a critical area of research, with recent studies highlighting the importance of minimizing or even avoiding ALND altogether due to its significant morbidity and limited overall survival benefits in specific cases [44, 45].

## 5. Conclusions

To our knowledge, this is the first study in order to evaluate the potential relationship between primary BC location and ALN metastasis using the OSNA assay. While no statistically significant differences were found between breast tumor location and SLN status, the number of metastatic ALNs, or the mean TTL, a trend toward a higher mean TTL was observed in tumors located in the INLs. These tumors exhibited pathologic features associated with biological aggressiveness and poorer prognosis.

Moreover, no significant differences in axillary staging were noted across breast tumor locations, regardless of whether superparamagnetic iron oxide or patent blue dye with radioisotope was used for SLN detection. This study highlights the need for further research to better understand the relationship between primary BC location and axillary nodal status. Such research could improve prognostic stratification and promote the development of tailored surgery and personalized adjuvant therapies. However, these findings should be interpreted with caution, and additional studies are necessary to confirm and expand upon these observations.

## Figures and Tables

**Table 1:** Clinical characteristics of the study group, comparing patients with primary tumors in EXL (external quadrants and axillary tail), INL (internal quadrants), and NAL (nipple and areola) locations.

Clinical characteristics	EXL	INL	NAL	*p* value
	*N* = 160	*N* = 58	*N* = 17	
Age, years				0.941^Ω^
Minimum	32	43	49	
Maximum	78	79	79	
Mean ± SD	59 ± 8.6	60 ± 8.5	60 ± 6.1	

Parity (%)				0.424^¥^
Nulliparity	8.8% (*n* = 14)	6.9% (*n* = 4)	0% (*n* = 0)	
Multiparity	66.0% (*n* = 106)	67.3% (*n* = 39)	92.9% (16)	

Breastfeeding, months				0.653^Ω^
Minimum	0	0	0	
Maximum	72	66	48	
Mean ± SD	8.8 ± 13.9	8.8 ± 13.9	11.9 ± 16.4	

Menopausal status (%)				0.767^¥^
Premenopausal	18.8% (*n* = 30)	17.2% (*n* = 10)	11.8% (*n* = 2)	
Postmenopausal	81.2% (*n* = 130)	82.8% (*n* = 48)	88.2% (*n* = 15)	

Age of menopause, years				0.171^Ω^
Minimum	37	46	44	
Maximum	60	56	52	
Mean ± SD	50 ± 4.1	49 ± 5.8	49 ± 2.9	

Postmenopausal hormone therapy (%)	3.8% (*n* = 6)	5.2% (*n* = 3)	0.0% (*n* = 0)	0.258^¥^

Smoker (%)	11.3% (*n* = 18)	3.4% (*n* = 2)	5.9% (*n* = 1)	0.167^¥^

Palpability (%)				0.062^¥^
Nonpalpable	59.4% (*n* = 95)	70.7% (*n* = 41)	82.4% (*n* = 14)	
Palpable	40.6% (*n* = 65)	29.3% (*n* = 17)	17.6% (*n* = 3)	

*Note:* ¥: chi-square test, Ω: one-way ANOVA.

Abbreviation: SD, standard deviation.

**Table 2:** Pathologic and molecular characteristics, comparing primary tumors in EXL (external quadrants and axillary tail), INL (internal quadrants), and NAL (nipple and areola) locations.

Pathologic characteristics	EXL	INL	NAL	*p* value
	*N* = 160	*N* = 58	*N* = 17	
Histologic type (%)				0.418^¥^
No special type (NST)	73.8% (*n* = 118)	81.0% (*n* = 47)	52.9% (*n* = 9)	
Invasive lobular	9.4% (*n* = 15)	6.9% (*n* = 4)	29.4% (*n* = 5)	
Invasive tubular	0.01% (*n* = 2)	0.0% (*n* = 0)	0.0% (*n* = 0)	
Ductal in situ	8.8% (*n* = 14)	8.6% (*n* = 5)	11.8% (*n* = 2)	

Tumor diameter, mm				0.641^Ω^
Minimum	2.0	5.0	2.0	
Maximum	60.0	62.0	50.0	
Mean ± SD	17.4 ± 10.2	15.2 ± 10.6	14.7 ± 10.2	

LVI (%)	5.6% (*n* = 9)	8.6% (*n* = 5)	5.9% (*n* = 1)	0.817^¥^

Grade				0.048^¥^
Grade 1 (%)	51.9% (*n* = 83)	46.6% (*n* = 27)	29.4% (*n* = 4)	
Grade 2 (%)	44.4% (*n* = 71)	41.4% (*n* = 24)	52.9% (*n* = 9)	
Grade 3 (%)	3.7% (*n* = 6)	12.0% (*n* = 7)	17.6% (*n* = 3)	

Ki67, %				0.798^Ω^
Minimum	<10	1	<10	
Maximum	90	40	30	
Mean ± SD	13 ± 12.1	14 ± 11.1	15 ± 8.6	

Intrinsic molecular subtype (%)				0.531^¥^
Luminal A	56.3% (*n* = 90)	58.6% (*n* = 34)	52.9% (*n* = 9)	
Luminal B	20.0% (*n* = 32)	17.2% (*n* = 10)	23.5% (*n* = 4)	
Luminal B/(HER2)+	11.9% (*n* = 19)	10.3% (*n* = 6)	5.9% (*n* = 1)	
HER2-enriched	0.0% (*n* = 0)	1.7% (*n* = 1)	5.9% (*n* = 1)	
Triple negative	1.9% (*n* = 3)	3.4% (*n* = 2)	0.0% (*n* = 0)	

*Note:* ¥: chi-square test, Ω: one-way ANOVA. Bold value denotes that the *p* value was statistically significant.

Abbreviations: HER2, human epidermal growth factor receptor-2; LVI, lymphovascular invasion; SD, standard deviation.

**Table 3:** Characteristics of the SLN, comparing patients with primary tumors in EXL (external quadrants and axillary tail), INL (internal quadrants), and NAL (nipple and areola) locations.

Characteristics of the SLN	EXL	INL	NAL	*p* value
	*N* = 160	*N* = 58	*N* = 17	
Technique for SLN detection (%)				0.905^¥^
Superparamagnetic iron oxide	45.6% (*n* = 73)	43.1% (*n* = 25)	41.2% (*n* = 7)	
Patent blue + radioisotope	54.4% (*n* = 87)	56.9% (*n* = 33)	58.8% (*n* = 10)	

SLN status (%)				0.937^¥^
Negative	83.1% (*n* = 133)	81.0% (*n* = 47)	82.4% (*n* = 14)	
Positive	16.9% (*n* = 27)	19.1% (*n* = 11)	17.6% (*n* = 3)	

Number of metastatic SLN				0.542^Ω^
1 metastatic SLN (%)	14.4% (*n* = 23)	17.2% (*n* = 9)	17.6% (*n* = 3)	
2 metastatic SLN (%)	2.5% (*n* = 4)	3.4% (*n* = 2)	0.0% (*n* = 0)	
Mean ± SD	0.2 ± 0.5	0.2 ± 0.5	0.35 ± 0.8	

OSNA result (%)				0.937^¥^
Negative (pN0)	83.1% (*n* = 133)	81.0% (*n* = 47)	82.4% (*n* = 14)	
Micrometastases (pN1mi)	6.9% (*n* = 11)	17.1% (*n* = 10)	17.6% (*n* = 3)	
Macrometastases (pN1)	10.0% (*n* = 16)	2.0% (*n* = 1)	0.0% (*n* = 0)	

TTL				0.570^Ω^
Minimum	170	280	360	
Maximum	430,000	420,000	470	
Mean ± SD	86,314 ± 140,522	96,536 ± 156,371	397 ± 64	

Note: ¥: chi-square test, Ω: one-way ANOVA.

Abbreviations: SD, standard deviation; SLN, sentinel lymph node.

## Data Availability

The clinical and pathologic data used to support the findings of this study are available from the corresponding author upon request.

## References

[1] Cardoso F., Kyriakides S., Ohno S. (2019). Early Breast Cancer: ESMO Clinical Practice Guidelines for Diagnosis, Treatment and Follow-Up. *Annals of Oncology*.

[2] Burstein H. J., Curigliano G., Loibl S. (2019). Estimating the Benefits of Therapy for Early-Stage Breast Cancer: The St. Gallen International Consensus Guidelines for the Primary Therapy of Early Breast Cancer 2019. *Annals of Oncology*.

[3] Gradishar W. J., Moran M. S., Abraham J. (2022). Breast Cancer, Version 3.2022, NCCN Clinical Practice Guidelines in Oncology. *Journal of the National Comprehensive Cancer Network*.

[4] Gradishar W. J., Anderson B. O., Abraham J. (2020). Breast Cancer, Version 3.2020, NCCN Clinical Practice Guidelines in Oncology. *Journal of the National Comprehensive Cancer Network*.

[5] Jimbo K., Kinoshita T., Suzuki J. (2013). Sentinel and Nonsentinel Lymph Node Assessment Using a Combination of One-Step Nucleic Acid Amplification and Conventional Histological Examination. *The Breast*.

[6] Yoon K. H., Park S., Kim J. Y. (2019). Is the Frozen Section Examination for Sentinel Lymph Node Necessary in Early Breast Cancer Patients?. *Annals of Surgical Treatment and Research*.

[7] Fougo J. L., Amendoeira I., Brito M. J. (2020). Sentinel Node Total Tumour Load as a Predictive Factor for Non-Sentinel Node Status in Early Breast Cancer Patients – The Porttle Study. *Surgical Oncology*.

[8] Shi F., Zhang Q., Liang Z., Zhang M., Liu X. (2017). One-Step Nucleic Acid Amplification Assay is an Accurate Technique for Sentinel Lymph Node Biopsy of Breast Cancer Patients: A Meta-Analysis. *British Journal of Cancer*.

[9] Bertozzi S., Londero A. P., Bulfoni M. (2022). One-Step Nucleic Acid Amplification System in Comparison to the Intraoperative Frozen Section and Definitive Histological Examination Among Breast Cancer Patients: A Retrospective Survival Study. *Frontiers in Oncology*.

[10] Tsujimoto M., Nakabayashi K., Yoshidome K. (2007). One-Step Nucleic Acid Amplification for Intraoperative Detection of Lymph Node Metastasis in Breast Cancer Patients. *Clinical Cancer Research*.

[11] Peg V., Espinosa-Bravo M., Vieites B. (2013). Intraoperative Molecular Analysis of Total Tumor Load in Sentinel Lymph Node: A New Predictor of Axillary Status in Early Breast Cancer Patients. *Breast Cancer Research and Treatment*.

[12] Kenny R., Wong G., Gould L., Odofin O., Bowyer R., Sotheran W. (2022). Can One-Step Nucleic Acid Amplification Assay Predict Four or More Positive Axillary Lymph Node Involvement in Breast Cancer Patients: A Single-Centre Retrospective Study. *Annals of the Royal College of Surgeons of England*.

[13] Ohi Y., Umekita Y., Sagara Y. (2012). Whole Sentinel Lymph Node Analysis by a Molecular Assay Predicts Axillary Node Status in Breast Cancer. *British Journal of Cancer*.

[14] Sohn V. Y., Arthurs Z. M., Sebesta J. A., Brown T. A. (2008). Primary Tumor Location Impacts Breast Cancer Survival. *The American Journal of Surgery*.

[15] Siotos C., McColl M., Psoter K. (2018). Tumor Site and Breast Cancer Prognosis. *Clinical Breast Cancer*.

[16] Zhang Y., Li J., Fan Y. (2019). Risk Factors for Axillary Lymph Node Metastases in Clinical Stage T1-2N0M0 Breast Cancer Patients. *Medicine (United States)*.

[17] Shah A., Haider G., Abro N. (2022). Correlation Between Site and Stage of Breast Cancer in Women. *Cureus*.

[18] Manjer J., Balldin G., Garne J. P. (2004). Tumour Location and Axillary Lymph Node Involvement in Breast Cancer: A Series of 3472 Cases from Sweden. *European Journal of Surgical Oncology*.

[19] Ji F., Xiao W. K., Yang C. Q. (2019). Tumor Location of the Central and Nipple Portion is Associated With Impaired Survival for Women With Breast Cancer. *Cancer Management and Research*.

[20] Song X., Zhang Q. (2020). The Poor Prognosis of Lower-Inner Quadrant Breast Cancer in Patients Who Received Neoadjuvant Chemotherapy. *Annals of Palliative Medicine*.

[21] Sarp S., Fioretta G., Verkooijen H. M. (2007). Tumor Location of the Lower-Inner Quadrant is Associated With an Impaired Survival for Women With Early-Stage Breast Cancer. *Annals of Surgical Oncology*.

[22] Altundag K. (2017). Molecular Subtypes and Lower Inner Quadrant Tumors in Breast Cancer: Debate is Still Ongoing. *Clinical Breast Cancer*.

[23] Paredes P., Vidal-Sicart S., Zanón G. (2005). Clinical Relevance of Sentinel Lymph Nodes in the Internal Mammary Chain in Breast Cancer Patients. *European Journal of Nuclear Medicine and Molecular Imaging*.

[24] Patani N. R., Dwek M. V., Douek M. (2007). Predictors of Axillary Lymph Node Metastasis in Breast Cancer: A Systematic Review. *European Journal of Surgical Oncology*.

[25] Phung M. T., Tin Tin S., Elwood J. M. (2019). Prognostic Models for Breast Cancer: A Systematic Review. *BMC Cancer*.

[26] Yun S. J., Sohn Y. M., Seo M. (2017). Risk Stratification for Axillary Lymph Node Metastases in Breast Cancer Patients: What Clinicopathological and Radiological Factors of Primary Breast Cancer can Predict Preoperatively Axillary Lymph Node Metastases?. *Ultrasound Quarterly*.

[27] Voogd A. C., Coebergh J.-W. W., Driel O. J. R. v. (2000). The Risk of Nodal Metastases in Breast Cancer Patients With Clinically Negative Lymph Nodes: A Population-Based Analysis. *Breast Cancer Research and Treatment*.

[28] Bevilacqua J. L. B., Cody H. S., MacDonald K. A., Tan L. K., Borgen P. I., Van Zee K. J. (2002). A Model for Predicting Axillary Node Metastases Based on 2000 Sentinel Node Procedures and Tumour Position. *European Journal of Surgical Oncology*.

[29] Halverson K. J., Perez C. A., Myerson R., Levy J., Tucker G. (1994). halverson1994. *American Journal of Clinical Oncology: Cancer Clinical Trials*.

[30] Fein D. A., Fowble B. L., Hanlon A. L. (1997). Identification of Women With T1-T2 Breast Cancer at Low Risk of Positive Axillary Nodes. *Journal of Surgical Oncology*.

[31] Maibenco D. C., Weiss L. K., Pawlish K. S., Severson R. K. (1997). Presented at the 20th Annual San Antonio Breast Cancer Symposium.

[32] Velanovich V., Szymanski W. (1998). Lymph Node Metastasis in Breast Cancer: Common Prognostic Markers Lack Predictive Value. *Annals of Surgical Oncology*.

[33] Tanis P. J., Nieweg O. E., Valdés Olmos R. A., Kroon B. B. (2001). Collective Review Anatomy and Physiology of Lymphatic Drainage of the Breast From the Perspective of Sentinel Node Biopsy.

[34] Leong S. P., Pissas A., Scarato M. (2022). The Lymphatic System and Sentinel Lymph Nodes: Conduit for Cancer Metastasis. *Clinical and Experimental Metastasis*.

[35] Yang J., Yang Q., Mukherjee A., Lv Q. (2021). Distance Between the Tumour and Nipple as a Predictor of Axillary Lymph Node Involvement in Breast Cancer. *Cancer Management and Research*.

[36] Vrana D., Lukesova L., Gatek J. Predictive Parameters for Internal Mammary Node Drainage in Patients With Early Breast Cancer.

[37] Byrd D. R., Dunnwald L. K., Mankoff D. A. (2001). Internal Mammary Lymph Node Drainage Patterns in Patients With Breast Cancer Documented by Breast Lymphoscintigraphy.

[38] Fowble B., Hanlon A., Freedman G. (2000). PII S0360-3016(00)00526-5 Clinical Investigation Breast Internal Mammary Node Irradiation Neither Decreases Distant Metastases Nor Improves Survival in Stage I and II Breast Cancer.

[39] Cong B. B., Cao X. S., Cao L. (2017). Internal Mammary Lymph Nodes Radiotherapy of Breast Cancer in the Era of Individualized Medicine. *Oncotarget*.

[40] Wang W., Qiu P., Li J. (2022). Internal Mammary Lymph Node Metastasis in Breast Cancer Patients Based on Anatomical Imaging and Functional Imaging.

[41] Simon K., Dodelzon K., Drotman M. (2019). Accuracy of Synthetic 2D Mammography Compared With Conventional 2D Digital Mammography Obtained With 3D Tomosynthesis. *American Journal of Roentgenology*.

[42] Bin L., Huihui Y., Weiping Y., Changyuan W., Qinghong Q., Weiyu M. (2019). Value of Three-Dimensional Ultrasound in Differentiating Malignant from Benign Breast Tumors: A Systematic Review and Meta-Analysis. *Ultrasound Quarterly*.

[43] Teshome M., Wei C., Hunt K. K., Thompson A., Rodriguez K., Mittendorf E. A. (2016). Use of a Magnetic Tracer for Sentinel Lymph Node Detection in Early-Stage Breast Cancer Patients: A Meta-analysis. *Annals of Surgical Oncology*.

[44] Douek M., Klaase J., Monypenny I. (2014). Sentinel Node Biopsy Using a Magnetic Tracer Versus Standard Technique: The SentiMAG Multicentre Trial. *Annals of Surgical Oncology*.

[45] Man V., Suen D., Kwong A. (2023). Use of Superparamagnetic Iron Oxide (SPIO) Versus Conventional Technique in Sentinel Lymph Node Detection for Breast Cancer: A Randomised Controlled Trial. *Annals of Surgical Oncology*.

